# Native cellulose nanofibrills induce immune tolerance *in vitro* by acting on dendritic cells

**DOI:** 10.1038/srep31618

**Published:** 2016-08-25

**Authors:** Sergej Tomić, Vanja Kokol, Dušan Mihajlović, Aleksandar Mirčić, Miodrag Čolić

**Affiliations:** 1 University of Defense, Medical Faculty of the Military Medical Academy, Institute for Medical Research, Belgrade, Serbia; 2 University of Maribor, Faculty of Mechanical Engineering, Institute for Engineering Materials and Design, Maribor, Slovenia; 3 University of Belgrade, Institute of Histology and Embryology, School of Medicine, Belgrade, Serbia; 4 University of Belgrade, Institute for Application of Nuclear Energy, Belgrade, Serbia

## Abstract

Cellulose nanofibrills (CNFs) are attractive biocompatible, natural nanomaterials for wide biomedical applications. However, the immunological mechanisms of CNFs have been poorly investigated. Considering that dendritic cells (DCs) are the key immune regulatory cells in response to nanomaterials, our aim was to investigate the immunological mechanisms of CNFs in a model of DC-mediated immune response. We found that non-toxic concentrations of CNFs impaired the differentiation, and subsequent maturation of human monocyte-derived (mo)-DCs. In a co-culture with CD4^+^T cells, CNF-treated mo-DCs possessed a weaker allostimulatory and T helper (Th)1 and Th17 polarizing capacity, but a stronger capacity to induce Th2 cells and CD4^+^CD25^hi^FoxP3^hi^ regulatory T cells. This correlated with an increased immunoglobulin-like transcript-4 and indolamine dioxygenase-1 expression by CNF-treated mo-DCs, following the partial internalization of CNFs and the accumulation of CD209 and actin bundles at the place of contacts with CNFs. Cumulatively, we showed that CNFs are able to induce an active immune tolerance by inducing tolerogenic DCs, which could be beneficial for the application of CNFs in wound healing and chronic inflammation therapies.

Nanocellulose has become a promising nanomaterial for various technical, nutritional, pharmaceutical and biomedical applications^1^,^2^. Out of the three main types of nanocellulose materials (bacterial nanocellulose (BNCs), cellulose nanocrystals (CNCs) and cellulose nanofibrills (CNFs)), only CNFs possess a relatively low rigidity, thanks to the alternating crystalline (contributing to stiffness and elasticity) and amorphous cellulose structure (contributing to flexibility and plasticity)^2^, which significantly widens their biomedical applications. Due to their outstanding physical properties, special surface chemistry and good biocompatibility, CNFs have been explored as scaffolds for tissue-engineering^2^,^3^, 3D matrices for growth of various cells^4^, hemodialysis membranes^5^, antimicrobial nanomaterials^6^ and in long-lasting sustained drug-delivery systems^7^. More interestingly, native CNFs were shown as quite promising platforms for skin tissue repair systems with wound healing properties. In this sense, CNFs were shown to support the growth of stem cells^8^ and they do not induce cytotoxicity and inflammation^9^, which is crucial for their long bio-persistence in the organism. Moreover, it was shown that CNFs induce anti-inflammatory effects on human keratinocytes *in vitro*^10^ and can suppress the inflammatory bowel disease in a mouse model^11^. These properties of CNFs are quite promising for their application in wound healing and chronic inflammation therapies. However, the immunological mechanism of CNFs’ anti-inflammatory effects remained almost completely unexplored.

The key innate immune cells regulating the immune response to various nanomaterials are dendritic cells (DCs)^12^. DCs are able to recognize nanomaterials via innate immunity receptors, and respond differently depending on the receptor/signaling engaged, as well as nanomaterials’ composition, structure, size, charge, functional groups and other factors, as shown by many papers^13^, including our own^14^,^15^. The immunogenic response of DCs includes the up-regulation of MHC molecules, co-stimulators, and pro-inflammatory cytokines production, followed by the activation of naïve T cells differentiation towards different effectors T cells^16^,^17^. In contrast, DCs are also able to induce immune tolerance by expressing inhibitory molecules, such as immunoglobulin-like transcript (ILT)3, ILT4^18^ and indolamine dioxygenase (IDO)-1^19^ and anti-inflammatory cytokine IL-10, which enables them to induce anti-inflammatory and regulatory T cell (Treg) populations^20^. These properties candidate DCs as the key targets for the therapy of cancer, autoimmune diseases^21^, chronic inflammatory conditions^22^, as well as for the induction of implant acceptance response^23^ and wound healing^24^. However, the effects of CNFs on DC-mediated immune response have not been investigated to date.

Previous studies carried out on different models of macrophages suggested that CNFs^25^,^26^ and CNCs^27^ do not induce cytotoxicity and the production of pro-inflammatory cytokines. Additionally, nanocellulose films were able to suppress the lipopolysaccharide (LPS)-induced production of pro-inflammatory cytokines by THP-1 macrophages^28^. The only study investigating the effects of CNCs on human DCs in a 3D culture system, found neither a significant modulation of IL-8 and tumor necrosis factor (TNF)-α production by DCs, nor the internalization of CNCs by these DCs^27^. Our recent data suggested that CNFs do not cause acute cytotoxicity in human peripheral blood mononuclear cells (PBMCs) but can significantly inhibit the proliferation of PBMCs stimulated by phytohemaglutinin (PHA), down-regulate the production of T helper (Th)1 cytokines, IL-2 and interferon (IFN)-γ, and increase the production of IL-10^29^. Since the PHA-induced effects in this model depend exclusively on the presence of antigen presenting cells (APCs) (predominantly DCs) within the PBMC population^30^, we hypothesized that the anti-inflammatory effects of CNFs are mediated through the modulation of DCs. In particular, we wondered how CNFs modulate the differentiation, subsequent maturation and functions of DCs, including their allostimulatory capacity, Th polarizing functions and the ability to induce regulatory T cells, using human monocyte-derived (mo)-DCs as a model system. Besides, we assessed what potential mechanisms of CNFs are involved in their actions on these cells.

## Results

### Morphological and surface analysis of CNFs

**Figure 1:**
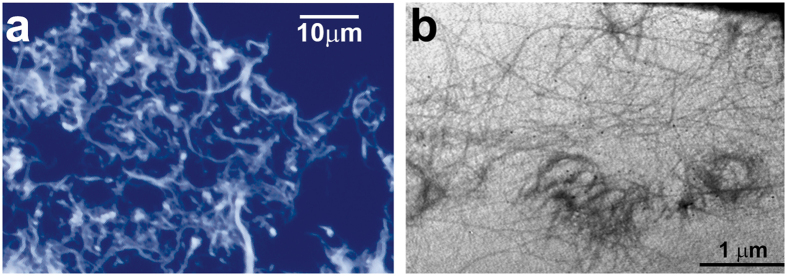
Morphology of CNFs. (**a**) Light microscopy representation of Calcofluor-stained CNFs dispersed on glass microscopic slides. (**b**) TEM micrograph of CNFs.

CNFs with a high content of cellulose (95%) were prepared and characterized as previously described^29^,^31^. The ζ-potential of the CNFs with an average size of 33,165 ± 2,516 nm in phosphate buffered saline (PBS) was estimated to be −23.84 ± 6.54 mV, being attributed to partly dissociated terminal-located carboxylic (-COO-) acid groups. To observe the morphology of CNFs in the cell culture media prior to cell culture experiments, the samples were conditioned in the complete RPMI medium for 24 h and then prepared for the fluorescent microscopy and transmission electron microscopy (TEM) analysis, respectively (Fig. 1). Calcofluor white-stained CNFs, fluorescing brightly upon the excitation with UV-B light (330–380 nm), consisted of fine fibrils different in length and thickness, fibro-reticular structures, and small brightly stained dots sized up to 1 μm. More detailed TEM analysis revealed that such dots were formed by small bundles of cellulose fibrils, about 70 nm thick, forming irregularly shaped circles. The smallest non-branching fibrils observed by the TEM analysis were 10–40 nm thick, which is in accordance with our previous observation made by atomic force microscopy (AFM)^29^. This suggested that the morphology of CNFs is similar in the cell culture medium and in the physiological solution used in the previous AFM analysis.

### Cytotoxicity of CNFs in mo-DC cultures

To investigate the modulatory effects of CNFs on DC differentiation, maturation and functions, we used a model of human mo-DCs differentiated into immature DCs in the presence of granulocyte-macrophage colony stimulating factor (GM-CSF)/IL-4 for 5 days, and then matured with the standard pro-inflammatory cytokines cocktail (prostaglandin (PG)E2, IL-6, IL-1β and TNF-α)^32^, a combination of TLR3 and TLR4 agonist (Poly (I:C)/LPS)^33^, or TLR7/8 agonist (CL075) to potentiate Th1 polarizing capacity of DCs^34^, and a TLR1/2 agonist (Pam3Csk4) to stimulate DC-mediated Th2 response^35^. CNFs were added to mo-DC cultures during the differentiation phase, in two different concentrations of CNFs (100 and 500 μg/ml) that were not cytotoxic in our previous study^29^.

The cytotoxicity study suggested that neither concentration affected the viability and apoptosis of mo-DCs during their 5-day differentiation from monocytes. This was confirmed by PI/hypotonic solution staining of late apoptotic cells (Supplementary Fig. 1a,b), and by Annexin-V-fluorescein isothiocyanate (FITC)/propidium iodide (PI) staining (Supplementary Fig. 1c,d).

### Differentiation and maturation of mo-DCs are impaired in the presence of CNFs

**Figure 2:**
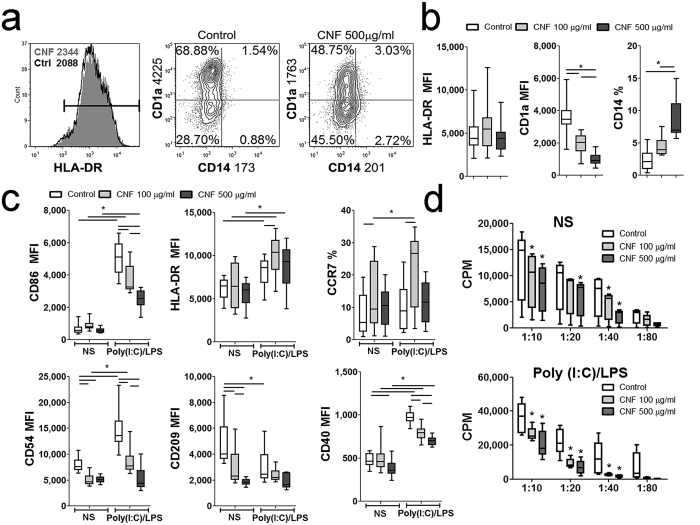
Effects of CNFs on differentiation, Poly (I:C)/LPS-induced maturation and allostimulatory capacity of mo-DCs. (**a**) The expression of HLA-DR, CD1a and CD14 was analyzed on immature DCs generated from monocytes in GM-CSF/IL-4-supplemented medium in the presence or absence of CNFs (Control) after 5 days. The results from one representative experiment, or (**b**) 8 experiments (mo-DC donors) are shown. (**c**) The phenotypic analysis of mo-DCs which were stimulated with Poly (I:C)/LPS, or were left non-stimulated (NS) for additional 2 days was carried out, and the results from 9 experiments are shown. (**d**) The proliferation in co-culture with either NS or Poly (I:C)/LPS-matured mo-DCs, and MACS purified CD4^+^T cells (1 × 10^5^ cells/well), carried out in different mo-DC-to-T cell ratios (1:10–1:80), was measured by 3H-thymidin incorporation assay after 5 days (cpm, counts per minute). The results from 8 different mo-DC/CD4 proliferation assays are shown. *p < 0.05 compared to control, or as indicated by line (Friedman test with Dunns posttest) NS - non-stimulated.

Even though not cytotoxic at the applied concentrations, CNFs significantly affected the differentiation of mo-DCs. Namely, control mo-DCs down-regulated CD14 expression completely and up-regulated CD1a expression. In contrast, mo-DCs differentiated with CNFs expressed significantly lower levels of CD1a and higher levels of CD14, and the effect was stronger with higher concentrations of CNFs (Fig. 2a,b). The expression of HLA-DR on immature mo-DCs was similar between the control and CNF-treated mo-DCs.

To evaluate whether subsequent maturation was affected, control and CNF-treated mo-DCs were stimulated with poly (I:C)/LPS (Fig. 2c), CL075 and Pam3Csk4 (Supplementary Fig. 2), or pro-inflammatory cytokines (IL-1β, TNF-α, IL-6, PGE2) (Supplementary Fig. 3) for 2 days. Immature mo-DCs were cultivated likewise, but without the maturational stimuli. When the latter were cultivated with a lower concentration of CNFs (100 μg/ml), mo-DCs expressed significantly higher levels of chemokine receptor (CCR)7. Immature mo-DCs treated with CNFs had a lower expression of CD54, CD209 (DC-SIGN) and CD40 compared to control immature mo-DCs (Fig. 2c).

Poly (I:C)/LPS up-regulated significantly the expression of CD86, HLA-DR, CD40 and CD54, and down-regulated the expression of CD209 by control mo-DCs, whereas CCR7 expression remained unaffected (Fig. 2c). CNF-treated mo-DCs up-regulated the expression of CD86, CD54 and CD40 upon Poly (I:C)/LPS treatment, albeit to a significantly lesser extent compared to corresponding control mo-DCs. Mo-DCs treated with CNF (100 μg/ml) expressed higher levels of CCR7 and HLA-DR upon Poly (I:C)/LPS stimulation, compared to corresponding mature mo-DCs. Stimulation of CNF-treated mo-DCs with Poly (I:C)/LPS did not additionally down-regulate CD209 expression. Since CNFs could potentially lower the active concentration of these stimulators for mo-DCs, CNFs (500 μg/ml) were conditioned with Poly (I:C)/LPS for 24 h and then centrifuged. The supernatant of CNF-conditioned medium had a similar stimulatory effects on mo-DCs as control conditioned Poly (I:C)/LPS medium (data not shown), suggesting that CNFs did not sequester a significant amount of Poly (I:C)/LPS.

The impaired differentiation and maturation capacity of CNF-treated mo-DCs was reflected in their weaker allostimulatory capacity as well. Namely, both immature and Poly (I:C)/LPS-matured mo-DCs differentiated in the presence of CNFs possessed a significantly lower capacity to stimulate the proliferation of allogeneic CD4^+^T cells compared to corresponding control mo-DCs (Fig. 2d). The maturation and allostimulatory capacity of mo-DCs treated with CNFs (500 μg/ml) was also lower when other TLR agonists (CL075 and Pam3Csk4) or proinflammatory cytokines cocktail, were used instead of Poly (I:C)/LPS, although some differences between the effects of these stimuli were observed (Supplementary Figs 2 and 3).

### Th polarization capacity of mo-DCs differentiated in the presence of CNFs

In addition to the stimulation of T cell proliferation, a key role of DCs is to shape the effector T cell response through the polarization of naïve CD4^+^ T cell differentiation, which is enabled predominantly via cytokines secreted by DCs^16^,^17^. To observe how CNFs affect the polarization capacity of mature mo-DCs, supernatants of Poly (I:C)/LPS-treated mo-DC cultures were collected and the levels of IL-12, IL-23, IL-10, TNF-α, IL-6, IL-27 and transforming growth factor (TGF)-β were measured.

**Figure 3:**
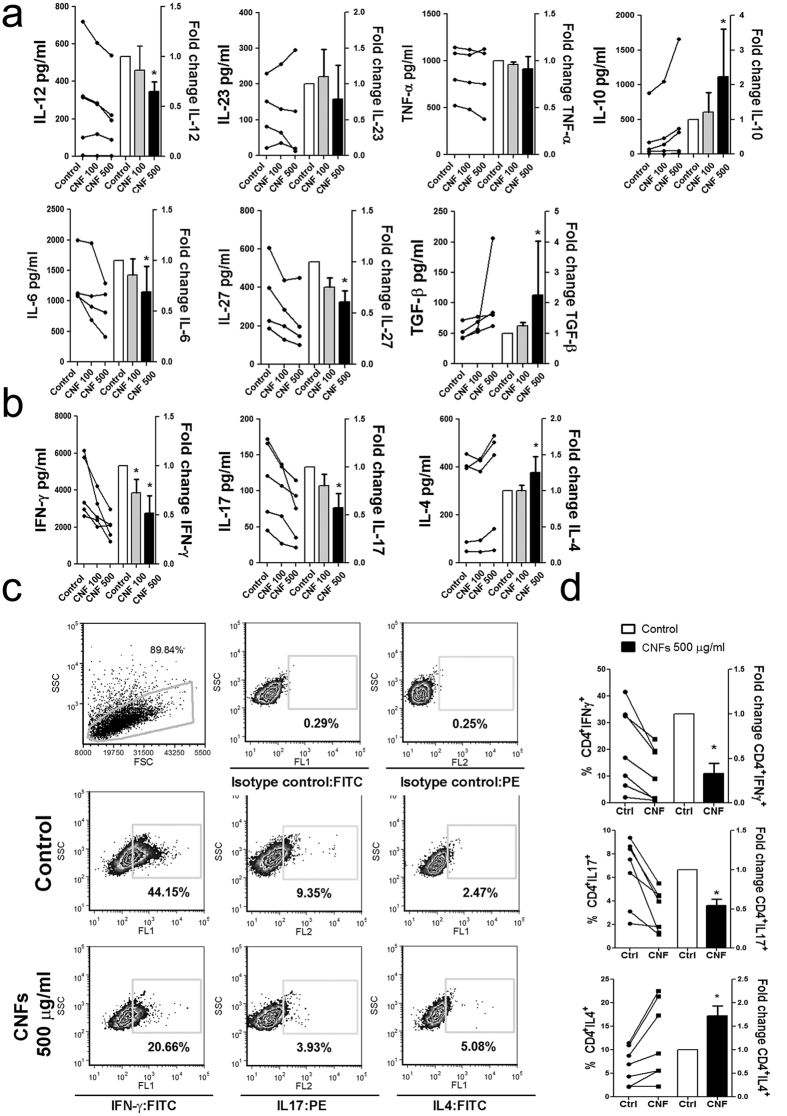
Effects of CNFs on Poly (I:C)/LPS-induced production of cytokines and Th polarization capacity of mo-DCs. (**a**) The levels of IL-12p70, IL-10 (from 5 different mo-DC cultures), IL-23, TNF-α, IL-6, IL-27 and TGF-β (4 different mo-DC cultures) were determined by enzyme-linked immunosorbent assay (ELISA) in the cultures of CNF-treated and control mo-DCs matured with Poly (I:C)/ LPS for 2 days. The levels of cytokines detected in an experiment with mo-DCs from one donor are linked by lines, and the summarized results are shown beside as mean fold change ± SD. (**b**) The levels of IFN-γ, IL-4 and IL-17 detected in each of the 5 different Poly (I:C)/LPS-matured mo-DCs/CD4^+^T cell co-cultures, carried out in 1:20 mo-DC-to-T cell ratio for 5 days, are linked by lines and the summarized results are shown beside as mean fold change ± SD; *p < 0.05 compared to control (Friedman test with Dunns posttest). (**c**) The data from one experiment on intracellular staining of CD4^+^T cells co-cultivated with mo-DCs, with high IFN-γ/IL-4 ratio in control cultures, are shown (see Supplementary Fig. 4b for the results on CD4^+^ T cell donor with low IFN-γ/IL-4 ratio in control cultures). (**d**) The percentages of IFN-γ^+^, IL-4^+^ and IL-17^+^CD4^+^ detected in each of the 7 different experiments are shown, and the summarized results are shown beside as mean fold change ± SD *p < 0.05 compared to control (Wilcoxon signed rank test).

It was found that the low concentration of CNFs (100 μg/ml) did not affect the production of cytokines by Poly (I:C)/LPS-treated mo-DCs. In contrast, the high concentration of CNFs (500 μg/ml) significantly impaired the capacity of these mo-DCs to produce IL-12p70, IL-6 and IL-27, but augmented their ability to produce IL-10 and TGF-β (Fig. 3a). The levels of IL-23 and TNF-α were not significantly affected. Similar effects of CNFs on the IL-10 production capacity of mo-DCs were observed after CL075 and Pam3Csk4 stimulations. As with Poly (I:C)/LPS stimulation, the IL-12- and IL-6 production capacities of CNF-treated mo-DCs upon CL075 stimulation were significantly impaired compared to corresponding control mo-DCs, whereas the levels of IL-12 and IL-6 in Pam3Csk4-treated cultures were not changed significantly (Supplementary Fig. 4a).

To investigate whether the change in the capacity of mo-DCs to produce these cytokines affects their Th polarization capability, Poly (I:C)/LPS matured mo-DCs were co-cultivated with purified allogeneic CD4^+^ T cells (1:20 cell-to-cell ratio), and the levels of IFN-γ, IL-4, and IL-17A cytokines as well as the percentages of IFN-γ^+^, IL-4^+^ and IL-17^+^CD4^+^ T cells were determined in co-cultures. Significant negative correlation between the percentages of IFN-γ^+^CD4^+^ and IL-4^+^CD4^+^ T cells existed in different co-cultures with control Poly (I:C)/LPS-treated DCs (R = −0.9286, p = 0.0067, n = 7, Spearman test). Irrespective of that, mo-DCs differentiated with CNFs had a significantly impaired capacity to induce IFN-γ and IL-17 production by CD4^+^ T cells (Fig. 3b). Intracellular staining confirmed a significantly lower percentage of CD4^+^IFN-γ^+^ and CD4^+^IL-17^+^ T cells in co-cultures with CNF-differentiated mature mo-DCs (Fig. 3c,d).

In contrast, mo-DCs differentiated in the presence of higher doses of CNFs (500 μg/ml) had a much stronger Th2 polarization capacity, since increased IL-4 levels and the percentages of CD4^+^IL-4^+^ T cells were detected in co-cultures with CNF-treated mo-DCs, compared to co-cultures with control Poly (I:C)/LPS-treated mo-DCs (Fig. 3b–d). Similar results on the Th polarization capacity of CNF-treated mo-DCs were obtained when CL075 were used for the maturation of these cells. In contrast, Pam3Csk4-stimulated CNF-treated mo-DCs had an increased capacity to induce IL-4^+^CD4^+^T cells, and a low capacity to induce IFN-γ^+^ and IL17^+^ CD4^+^T cells which was not significantly different form the Pam3Csk4-stimulated control mo-DCs (Supplementary Fig. 4b,c).

### Tolerogenic properties of mo-DCs differentiated in the presence of CNFs

**Figure 4:**
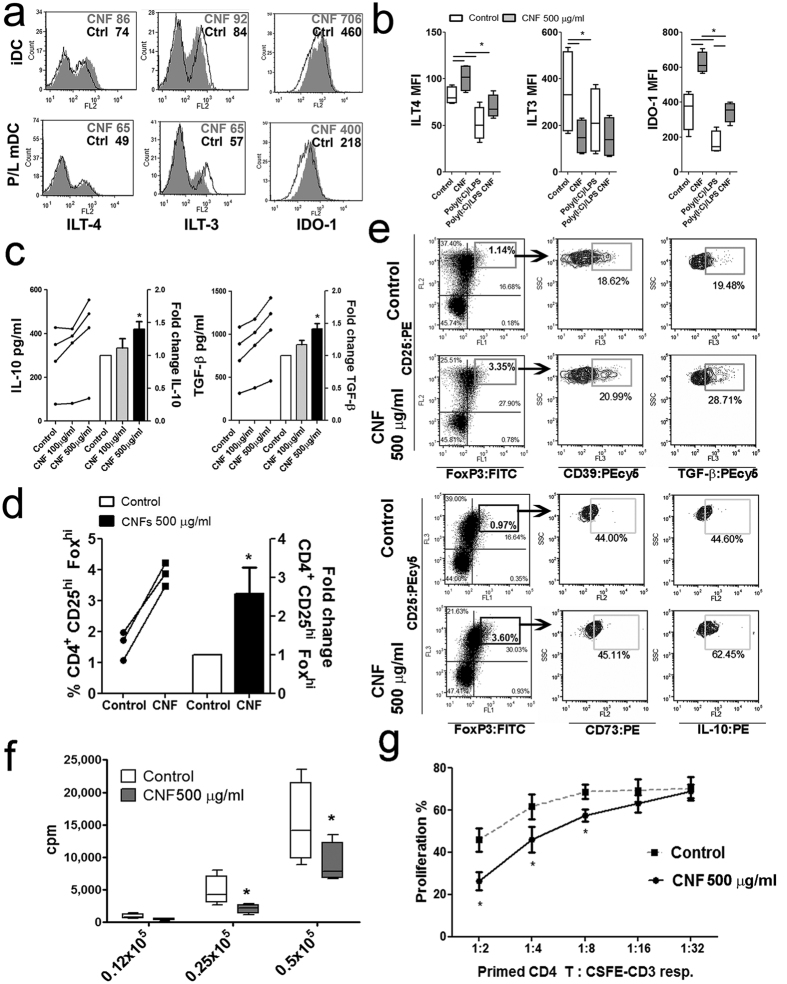
Tolerogenic functions of CNF-treated mo-DCs. (**a**) The expression of ILT4, ILT3 and IDO-1 by CNF (500 μg/ml)-treated and control mo-DCs after the 48h-maturation with Poly (I:C)/LPS. The data from a representative experiment or (**b**) 5 independent experiments are shown. (**c**) The levels of IL-10 and TGF-β detected in each of the 4 different priming assays with Poly (I:C)/LPS-matured mo-DCs and CD4^+^T, carried out in 1:50 mo-DC-to-T cell ratio, are linked by lines and the summarized results are shown beside as mean fold change ± SD. (**d**) The percentages of CD4^+^CD25^hi^FoxP3^hi^ T cells detected in each of the 3 priming assays are shown, and the summarized results are shown beside as mean fold change ± SD. (**e**) A representative phenotypic analysis of CD4^+^T cells primed with control or CNF-treated mo-DCs is shown, out of 3 with similar results. During the analysis, each sample was stained with CD25:PE/FoxP3:FITC/CD39PEcy5, CD25:PE/FoxP3:FITC/TGF-β:PEcy5, CD25:PEcy5/FoxP3:FITC/CD73PE and CD25:PEcy5/FoxP3:FITC/IL-10:PE. (**f**) The proliferation of CD4^+^T cells primed with Poly (I:C)/LPS and then re-stimulated with CD3/CD28 antibodies was measured by 3H-thymidin incorporation assay after 5 days. The data from 4 different proliferation assays are shown. (**g**) The results on the proliferation of CFSE-labeled allogenic CD3^+^T cells co-cultivated with non-labeled CD4^+^T cells (1:2–1:32 CD4^+^T:CD3^+^T cell ratios) primed previously with either control or CNF-treated mo-DCs, are shown as mean ± SD of 3 suppression assays carried out with CD4^+^T primed with 3 different mo-DCs donors (see a representative experiment in Supplementary Fig. 5). *p < 0.05 compared to corresponding control or as indicated (Friedman test with Dunns posttest).

Mo-DCs differentiated with CNFs (500 μg/ml) possessed a lower allostimulatory capacity and a higher Th2 polarizing capability, which points to their increased anti-inflammatory/tolerogenic capacity^18^,^19^,^20^. Therefore, we assessed the expression of tolerogenic markers (ILT-3, ILT-4 and IDO-1) by immature and Poly (I:C)/LPS-matured mo-DCs after 48 h cultures (Fig. 4a,b).

CNF-differentiated immature mo-DCs had a significantly lower expression of ILT-3 then control immature mo-DCs. Poly (I:C)/LPS treatment down-regulated ILT-3 expression by control mo-DCs, but not by CNF-differentiated mo-DCs. In contrast, CNF-differentiated immature mo-DCs possessed significantly higher expression of ILT-4 compared to control mo-DCs. Poly (I:C)/LPS-treatment suppressed significantly ILT-4 expression on control mo-DCs, but not on CNF-differentiated mo-DCs. A similar phenomenon, although more pronounced, was observed for IDO-1 expression in mo-DCs.

To prove that CNF-differentiated mo-DCs are able to induce tolerogenic T cells, CD4^+^ T cells were primed with either CNF-treated or control mature mo-DCs. Supernatants of CNF-differentiated mature mo-DC/CD4^+^T cell co-cultures, contained significantly higher levels of IL-10 and TGF-β compared to control mo-DC/CD4^+^T cell co-cultures (Fig. 4c). Additionally, the analysis of Tregs according to their high expression of CD25 and forkhead box transcription factor (Fox)P3^36^, confirmed a significantly higher percentage of CD4^+^CD25^hi^FoxP3^hi^ Treg cells in co-culture with CNF-treated mo-DCs, compared to corresponding control mo-DCs (Fig. 4d,e). In addition to the increased percentage of Tregs, CD4^+^T cells primed with CNF-treated mo-DCs expressed higher levels of IL-10 and TGF-β within the CD25^hi^FoxP3^hi^ population of CD4^+^T cells, whereas the levels of other Treg cell molecules (CD73 and CD39) were not changed (Fig. 4e).

To evaluate whether such an increase in the percentage of Treg cells have a functional significance, CD4^+^T cells primed with CNF-treated or control mo-DCs were collected and re-stimulated with anti-CD3 and anti-CD28 antibodies (Abs), to assess their capacity to proliferate to secondary stimulation. Additionally, these cells were co-cultured with the allogeneic carboxyfluorescein succinimidyl ester (CFSE)-labeled CD3^+^ T cells stimulated with anti-CD3/anti-CD28 Abs. The results suggested that, CD4^+^T cells primed with CNF-treated mature mo-DCs, are hypo-responsive to the re-stimulation with CD3/CD28 (Fig. 4f). Furthermore, those cells inhibited the polyclonal proliferation of responder T cells when added in 1:2, 1:4 and 1:8 (primed CD4^+^ T cells: responder CD3^+^T cells, respectively) ratio (Fig. 4g, Supplementary Fig. 5), which confirmed that CNF-treated mo-DCs are capable of inducing functionally suppressive CD4^+^ T cells.

### CNFs-mo-DCs interaction

**Figure 5:**
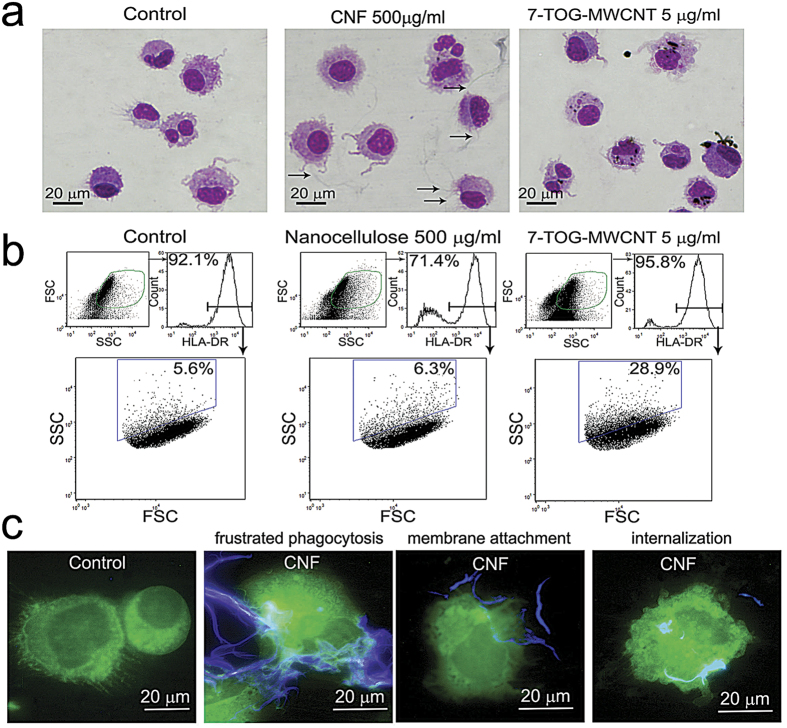
Internalization of CNFs by mo-DCs. (**a**) Mo-DCs differentiated in the presence or absence of CNFs were collected and the cytospins were stained with MGG. Immature control mo-DCs were additionally cultivated with 7-TOG-MWCNT, which was used as a positive control. Arrows mark the grayish CNFs on the samples. (**b**) The same samples were analyzed by flow cytometry after labeling of mo-DCs with anti-HLA-DR-Alexa 488 Ab. (**c**) Cytospins of mo-DCs cultivated with CNFs were stained in anti-HLA-DR-Alexa 488 and Calcofluor white, and then analyzed by the epi-fluorescent microscopy. Representative data and images are shown, collected from 5 different experiments.

To investigate how CNFs affect the differentiation and maturation of DCs, we studied the interactions between CNFs and mo-DCs in greater detail. May Grunwald Giemsa (MGG)-stained samples of mo-DCs differentiated in the presence of CNFs indicated that CNFs do not affect significantly the morphology of mo-DCs compared to control (Fig. 5a). Mo-DCs interacted with different sized CNFs, which were detected as grayish fibrils. There was no clear indication of accumulation of CNFs inside mo-DCs. In contrast, 7-oxo-8-thiaguanosine (7-TOG)-multiwall carbon nanotubes (MWCNT), which were used as a positive control^29^, accumulated in the cytoplasm of mo-DCs (Fig. 5a). The analysis of side-scatter parameters of HLA-DR^+^ mo-DCs in culture also suggested that CNF-treated mo-DCs do not internalize significantly CNFs, since they do not form much higher levels of intracellular vesicles compared to control mo-DCs (Fig. 5b), unlike 7-TOG-MWCNTs-treated mo-DCs.

Next, we analyzed the interaction between mo-DCs and CNFs by epi-fluorescent microscopy, after staining with anti-HLA-DR: Alexa 488 mAb and Calcofluor. The interaction of mo-DCs and CNFs predominantly depended on the thickness and length of CNFs (Fig. 5c). Mo-DCs adhered to the large branched parts of CNFs which are several hundred micrometers long (Fig. 5c and Supplementary Fig. 6). The smaller branches thereby seemed to be partially internalized by mo-DCs, and the process resembled the frustrated phagocytosis. CNFs, up to 30 μm in length, were detected either attached to the cell membrane, as concluded according to the invaginations of mo-DC membranes at the places of mo-DC-CNF interactions, or completely internalized by mo-DCs.

**Figure 6:**
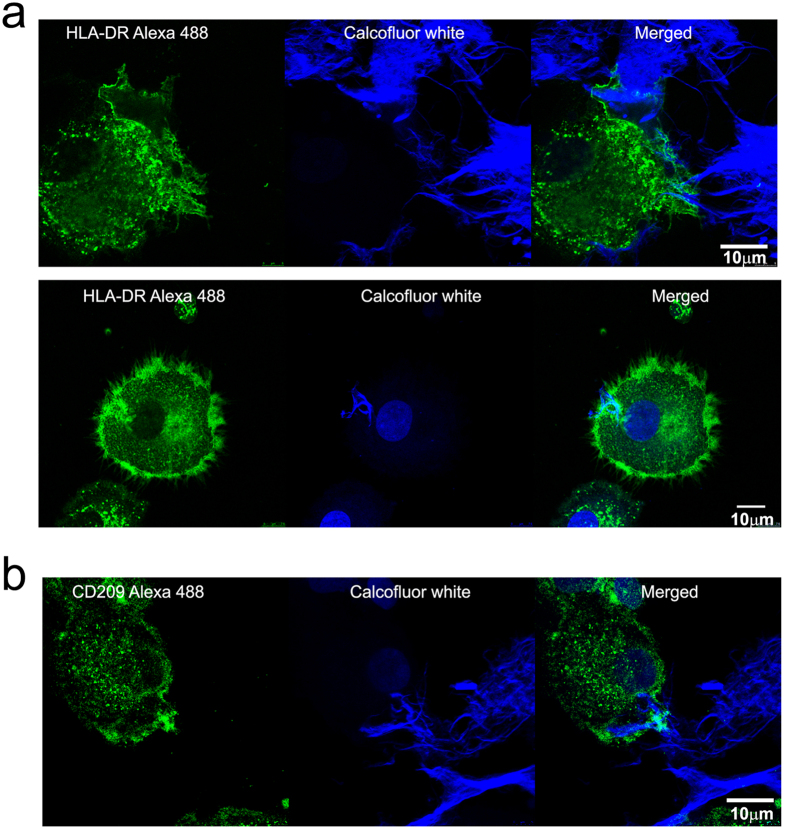
Analysis of CNFs internalization by mo-DCs with confocal microscopy. Cytospins prepared after the culture of mo-DCs with CNFs were stained with (**a**) anti-HLA-DR Alexa 488 or (**b**) anti-CD209 Alexa 488 and Calcofluor white, as indicated. Z-stacks of corresponding samples are provided as Supplementary videos 1–3.

Since the epi-fluorescent microscopy picks up out-of-focus fluorescence, we applied confocal microscopy analysis with Z-scanning, which confirmed that CNFs were internalized completely or partially by HLA-DR^+^ mo-DCs (Fig. 6). The majority of mo-DCs attached to the large branched CNFs and their membrane was wrapped around the ends of cellulose fibrils (Supplementary movies 1 and 2). Interestingly, it was revealed that CD209 expression was particularly accumulated on the parts of mo-DCs’ membranes that interact with CNFs (Fig. 6b, Supplementary video 3). However, the total CD209 expression was still significantly lower compared to control mo-DCs (data not shown). The expression of HLA-DR appeared increased on all outer membranes as well as at the place of interactions with CNFs.

**Figure 7:**
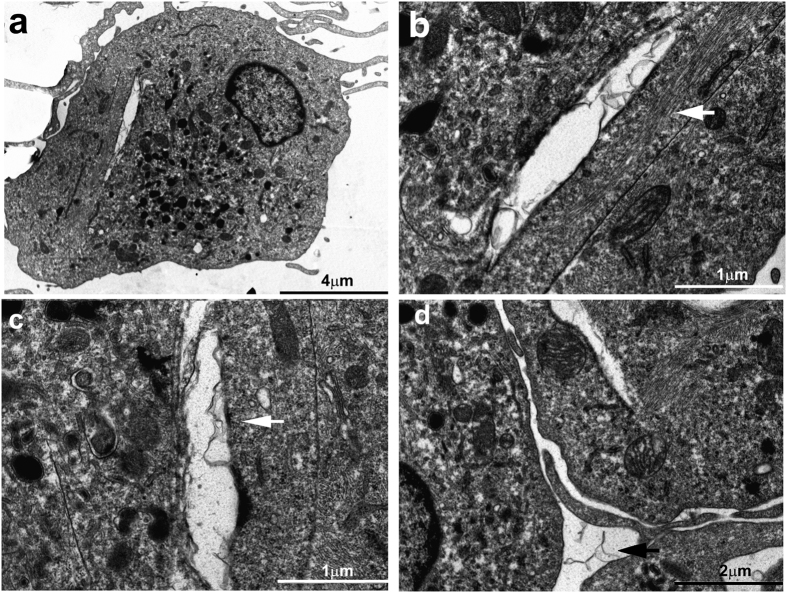
Analysis of CNFs internalization by mo-DCs with TEM. (**a**) CNF-treated mo-DC is shown, and slit-shaped endosome is magnified in (**b**). Similar structures were seen on other cells (**c**,**d**). Actin bundles were parallel (**b**) or perpendicular (**c**) to the longer axis of the endosome. (**d**) Extracellular CNFs interacting with the cell membrane are shown.

Finally, we analyzed the interaction between CNFs and DCs using TEM (Fig. 7). Long, slit-shaped, endosome-like structures containing CNFs were found in DCs. Similarly shaped structures were not found in control mo-DCs (data not shown). Since the TEM slices are very thin, the slit-shaped structures could represent the invagination of mo-DC membranes that are partially enwrapping CNFs. In line with that, the formation of actin bundles was detected in the vicinity of the slit-shaped endosomes, orientated parallel (Fig. 7b) or perpendicular (Fig. 7c) to the longer axis of the endosomes. Extracellular CNFs formed closed contacts with the outer membrane of mo-DCs (Fig. 7d).

## Discussion

CNFs were claimed to be quite a promising natural material with wide biomedical application potential^1^, including the preparation of wound dressings and the treatment of unwanted inflammations, due to their good biocompatibility and anti-inflammatory effects^10^,^29^. However, the immunoregulatory mechanisms of their anti-inflammatory effects *in vivo* and *in vitro* were not sufficiently addressed. In this paper we showed for the first time, that native CNFs are able to induce human tolerogenic DCs, which can down-regulate the Th1 and Th17-mediated response, expand Th2 cells, and induce Tregs. CNFs exhibit these properties at non-toxic concentrations. The lack of cytotoxicity of CNFs is in accordance with our previous study showing that CNFs are not toxic for L929 cells, rat thymocytes and human PBMCs even at 1 mg/ml^29^. The good cytocompatibility of CNFs was also described in the experiments with human monocytes, mouse macrophages^26^, human dermal fibroblasts^37^, and others^1^.

However, nanocellulose materials are not inert in the cell culture, as they were shown to possess anti-proliferative effects on different cell lines, especially if applied in high concentrations^38^. The anti-proliferative effects observed in the model of PHA-stimulated PBMCs^29^ could also include some of the specific immunological mechanisms through action on blood APCs. The previous studies on APCs/CNF interactions^25^,^26^,^27^,^28^,^29^, showed the lack of pro-inflammatory effects of CNFs, which is beneficial for their application in wound healing and the resolution of inflammation^39^. However, both the resolution of inflammation and wound healing process require specific anti-inflammatory mechanisms. In line with this, we showed that CNFs (250 μg/ml–1 mg/ml) down-regulate the production of pro-inflammatory Th1 cytokines, IL-2 and IFN-γ, Th17 cytokine IL-17A, but increase the production of anti-inflammatory cytokine IL-10, as well as IL-6^29^. IL-6, although considered a pro-inflammatory Th2 cytokine, is also crucial for the wound healing process^40^. Here we showed for the first time that the anti-inflammatory actions of CNFs can be mediated by the induction of tolerogenic DCs.

CNFs impaired the up-regulation of CD1a during the differentiation of DCs from monocytes. This significantly lowered the ratio of CD1a^+^ over CD1a^−^ mo-DCs in the CNF-treated mo-DC population. It was shown that CD1a^+^ mo-DCs, similar to the murine CD8α^+^ DC subset, produce a significant amount of IL-12 and can polarize naïve CD4^+^T cells to a Th1 phenotype, unlike the CD1a^−^ DC subset^41^. On the other hand, an increase in the frequency of CD1a^−^ mo-DCs was shown to correlate with increased production of IL-10 by these cells^42^, which is in line with our results. Additionally, CNF-treated mo-DCs exhibited an impaired phenotypic maturation, irrespective of the stimulation used. The effects of CNFs on the co-stimulatory molecules expression correlated with a lower allostimulatory capacity of CNF-treated mo-DCs in co-culture with CD4^+^ T cells. Therefore, impaired maturation and functions of CNF-treated mo-DCs were most probably a consequence of their altered differentiation pattern, rather than a non-specific sequestration of maturation agents to the CNFs. This was confirmed by comparing the pro- maturating effects of CNF-conditioned medium and control medium which contained Poly (I:C)/LPS. Such experiments also suggested that no hydrophilic active substances were released in mo-DC cultures from CNFs. Interestingly, the lower concentration of CNFs up-regulated the expression of HLA-DR and CCR7 by mo-DCs treated with Poly (I:C)/LPS or proinflammatory cocktail. Although these molecules are involved in the antigen presentation and migration of DCs, respectively^17^,^43^, they were also proven crucial for the induction of peripheral tolerance by these cells^43^,^44^, which is in line with the tolerogenic properties of CNF-treated mo-DCs. It is still not clear why only the lower concentration of CNFs had such an effect on mo-DC phenotype, so this requires additional investigation.

Considering the potential mechanism of CNFs action, we found that CNFs downregulated the expression of a C-type lectin CD209, while CD209 remained expressed particularly on the places of interaction with CNFs. The ligation of CD209 during the differentiation of DCs was shown to lead to an abrogated expression of CD1a, downregulation of CD209 and a low co-stimulatory expression upon maturation. Furthermore, those DCs possessed lower allostimulatory and Th1 polarizing capacity, and produced higher levels of IL-10^45^. Even the natural CD209 ligands were shown to induce DCs with an immature phenotype, increased Th2 polarizing capacity and down-regulated CD209 expression upon their internalization^46^,^47^. However, it is not known whether CD209 ligands prepared in a formulation that cannot be internalized by DCs, are also able to down-regulate CD209 expression and induce such effects via similar signaling mechanisms. If they are, it could be possible that the effects of CNFs are mediated by a direct interaction between CD209 and CNFs. The ligation of CD209 by CNFs is quite possible since, beside the cellulose (95%), CNFs used in this study contain up to 4.75% of hemicellulose, an amorphous branching heteropolymer containing Xylose-β(1,4)- Mannose- β(1,4) -Glucose- α (1,3)- Galactose. Crystallographic data of CD209 and its ligands suggested that this receptor recognizes several types of mannose- and fucose- containing branching glycans^48^. In line with this, our preliminary experiments suggested that CNFs (500 μg/ml) with a lower hemicellulose content (2.5%), possess lower inhibitory effects on the differentiation and maturation of mo-DCs, compared to CNFs used in this study. Therefore, this promising hypothesis deserves further testing, as it could open additional perspectives for the development of tolerance-inducing mannose-containing polymers.

Another key finding in this study was that CNFs-treated mo-DCs had a lower Th1- and Th17-, and an increased Th2-polarization capacity. The former could be explained by a reduced capacity of CNF-treated mo-DCs to produce Th1-polarizing cytokines IL-12 and IL-27^16^. Additionally, IL-10 is known to impair IL-12p70-induced production of IFN-γ by Th1 cells^49^, as well as the production of IL-17 by Th17 cells^50^. TGF-β inhibits Th1 development and IFN-γ production directly, whereas in the presence of IL-6 it promotes IL-17 production^51^. Therefore, a reduced IL-6/TGF-β production ratio by CNF-treated mo-DCs, could also explain their diminished Th17-polarizing capacity despite relatively constant IL-23 production, and an increased Tregs induction capacity. In a classical model, the down-regulation of IL-12 and Th1 cells is a default pathway for the induction of Th2 cells. Alternative models for Th2 induction by DCs include Syk-mediated signaling upon the activation of C-lectin type receptors, such as CD209, which is followed by down-regulation of IL-12, and up-regulation of IL-10 and Notch ligands Jagged 1 and Jagged 2^45^,^46^,^47^,^52^. Therefore, an increased Th2 polarization capacity of CNF-treated mo-DCs could be a consequence of the activation of either classical or alternative pathway for the Th2 mode.

Recent data suggested that the increased production of IL-10 and MHC-class II^44^, as well as TGF-β^53^ by DCs are required for the induction of different populations of Tregs. CNF-treated mo-DCs were indeed able to induce more CD4^+^CD25^hi^FoxP3^hi^ cells then control mo-DCs. CD4^+^CD25^hi^FoxP3^hi^ Tregs cells also expressed CD39 and CD73, which are involved in the adenosine-mediated suppression by Tregs^54^. Besides, CNF-treated mo-DCs potentiated both TGF-β and IL-10 within Tregs, and increased the levels of these cytokines in co-culture with CD4^+^T cells. The primed CD4^+^T cells were both hypo-responsive and suppressive for the allogeneic T cells. The suppressive effects of the cells primed with CNF-treated mo-DCs were probably a result of both increased percentage and activity of CD4^+^CD25^hi^FoxP3^hi^ Tregs, but also other functionally suppressive CD4^+^T cells (such as IL-10- and TGF-β-producing cells). It was shown that TGF-β- and IL-10-producing DCs are the key cells inducing Treg cells^53^, and one of the mechanism include ILT4/HLA-G interaction between DCs and T cells, respectively^18^. These results are in line with our findings on increased expression of ILT4 by CNF-treated mo-DCs. However, we did not find an increased expression of ILT3 by CNF-treated mo-DCs. It could be possible that ILT3 expression by CNF-treated mo-DCs is not necessary for their tolerogenic effects. In line with this, Penna *et al.*^55^ showed that the vitamin D3-induced expression of ILT3 on mo-DCs was not necessary for their capacity to induce Tregs. Furthermore, CNF-treated mo-DCs increased IDO-1 expression, which was described as a crucial factor contributing to the induction of Tregs by DCs in humans^19^. We showed previously that tolerogenic IDO-1^+^mo-DCs induced by mesenchymal stem cells, are able to induce functional CD4^+^CD25^+^FoxP3^+^IL-10^+^ Tregs, and that the blockage of IDO-1 in mo-DCs abrogated that effect^20^. Therefore, mo-DCs differentiated with CNFs indeed possess tolerogenic properties, which was strongly confirmed by both phenotypic assessment and by the functional analyses.

The only study investigating the effects of nanocellulose on human DCs in a 3D culture system found that human DCs do not internalize CNCs^27^. Our internalization studies revealed that mo-DCs closely interact with CNFs and enwrap them either partially or completely, depending on CNF size. These results confirm that CNFs provide a sufficient signal for mo-DCs to internalize them. According to the “tethering and tickling” model^56^, phagocytes first tether to the material and then require a tickling signal to internalize it. The tickling signal usually comes from C-type lectins, such as CD205, CD61, CD209 and others^56^. Signaling through these molecules, as well as TLRs, regulates the activity of small GTPases RhoA, Rac1 and Cdc42^46^, which are the central regulators of actin dynamics in cells, pre-requisite for internalization^57^. The actin formations around the slit-shaped endosomes observed in our experiments, resembling those found during the internalization process^58^, support the hypothesis that mo-DCs are trying to internalize CNFs. Aldinucci *et al.*^59^ showed recently that human mo-DCs adhere to the MWCNT-based scaffold during differentiation, which impairs their subsequent maturation induced by LPS, allostimulatory capacity, endocytosis, and the production of IL-12. The suggested mechanisms included the down-regulation of molecules involved in the actin reorganization, PI3K/AKT signaling and the formation of an immunological synapse in DCs. These results support the idea that actin cytoskeleton can act as the cell’s mechanosensitive receptor^60^. Although a similar explanation for our results is plausible, it is hard to prove that the signals from CNFs are not coming through some surface receptors on DCs. In line with this, it has been demonstrated that some serum proteins tethered to a biomaterial can stimulate DCs to release IL-10 and to prime Th2 cells^61^. Therefore, it is of high interest to evaluate what kind of protein shell is being generated on the surface of CNFs upon its interaction with the cultivation media and what mechanism predominates in CNF-DC interaction.

In conclusion, here we provided evidence for the first time, that native CNFs can induce active immune tolerance by inducing differentiation of tolerogenic human DCs, which are able to down-regulate Th1 and Th17 cells, and up-regulate Th2 and Tregs *in vitro*. The mechanisms of these actions, most probably lie within the specific nanostructure of CNFs containing both cellulose and hemicellulose components, and their interaction with CD209 and actin filaments. Such properties of CNFs could prove quite beneficial for the treatment of exaggerated inflammatory conditions threatening to destroy host tissue, as well as for supporting of wound healing. Although these results on human immune cells are quite promising, it would be necessary to confirm similar mechanisms of CNFs on other subpopulations of DCs and in corresponding *in vivo* model systems, before their safe clinical application. Thereby, all the advantages and disadvantages of the humanized model systems^62^ should be taken into account.

## Methods

### Cellulose nanofibers

As we reported previously^29^, bleached dissolving cellulose from the Norway spruce (*Picea abies*) with a very high (95%) cellulose content, hemicelluloses (4.75%) and a low lignin content was supplied by Domsjo Fabriker AB, Sweden, and used as the starting material. Cellulose fibers (2wt%) were dispersed in distilled water by using the Silverson mechanical blender L4RT at 6,000 rpm for 15 min. CNFs were then isolated by mechanical fibrillation using an ultrafine grinder at 1,440 rpm for 30 min, followed by a high pressure homogenizer^31^. The chemical composition of the CNFs obtained was considered to be same as the raw material as no chemical treatments were carried out during the fibrillation process.

The ζ-potential and average size measurements of the CNFs were performed in a 0.01 M PBS by a Nano ZS ZEN 3600 using the 633 nm laser and a field of 40 V across the nominal electrode spacing of 16 mm. All measurements were carried out at 25 ± 1 °C using the refractive index (1.33 vs. 1.48) and viscosity (0.8872 vs. 1.48 cP) of water versus cellulose for data analysis, being performed with Malvern Zetasizer Software 7.02. The influence of the CNF concentration and sonication conditions (time and amplitude) of dispersions on the ζ-potential and average size was evaluated in order to ascertain the relevance and validity of the data. Finally, the samples being prepared as 0.05% w/v solutions in PBS and additionally diluted to 0.005% w/v with Milli-Q water, were sonicated for 2 min at 25% amplitude before ζ-potential analysis using a SONICS Vibra cell™ VCX 750 ultrasonic processor.

Prior to their use in cell cultures, CNFs were sterilized in an autoclave at 121 °C during 30 min, and then sonicated. The concentrations used in the cell cultures (100 or 500 μg/ml) were prepared in a complete RPMI supplemented with 2 mM L-glutamine, gentamicin, penicillin, streptomycin, 50 μM 2- mercaptoethanol, and 10% heat-inactivated fetal calf serum (FCS). The level of endotoxin in the highest concentration of CNFs, as determined by the Limulus Amebocyte Lysate (LAL) test, was 0.16 EU/ml.

The morphology of CNFs conditioned in the complete medium for 24 h was assessed by fluorescent microcopy and transmission electron microscopy (TEM). CNFs were then centrifuged onto microscopic slides, air-dried, and then stained with Calcofluor white (Ex: 355 nm, Em:433 nm) at 1:100 dilution in PBS, prior to the epifluorescence microscope analysis using a UV-2A filter. For the TEM analysis, CNFs were centrifuged in PBS, fixed with 2% glutaraldehyde and postfixed in 1% OsO4. After dehydration in graded alcohols, thin sections were embedded in Epon 812 and analyzed by TEM (Morgagni 268D).

### Cell cultures

PBMCs were obtained from the Buffy coats of 20 healthy volunteers, as described^29^. Informed consents were obtained from all subjects who provided blood samples. The experiments protocols were approved by the Ethical Board of the Military Medical Academy, University of Defense (permission date: September 12th, 2012 in Belgrade), and carried out in accordance with the Institutional guidelines. The monocytes were isolated from PBMCs using Monocyte Isolation kit (Myltenil Biotec, Bergisch Gladbach, Germany), following their cultivation in complete RPMI medium containing human recombinant GM-CSF (100 ng/ml, Leucomax) and human recombinant IL-4 (20 ng/m, Roche Diagnostics). CD4^+^T and CD3^+^ T cells were isolated from PBMCs by negative magnetic sorting, using a CD4^+^T cell kit and a Pan T cell isolation kit (Myltenil Biotec), respectively, and they were used as responders in co-culture experiments with mo-DCs and suppressive assays. The purity of monocytes and T cell populations was usually higher than 85%, as evaluated by flow cytometry analysis (6-Cube, Systemx Partec GmbH, Görlitz, Germany) (Supplementary Fig. 7a).

To assess the effects of CNFs on the differentiation of DCs, monocytes were cultivated in the presence or absence of CNFs (100 or 500 μg/ml) for 5 days in a complete RPMI medium supplemented with GM-CSF/IL-4. To induce the maturation of mo-DCs, the cell cultures were treated with a combination of TLR3 and TLR4 agonist, poly (I:C) (10 μg/ml, Sigma-Aldrich) and LPS from *E.coli* 0.222:B4 (100 ng/ml, Sigma-Aldrich), respectively, for 48 h. Additionally, mo-DCs were matured with a TLR7/8 agonist CL075 (Invivogen) at 10 μM which predominantly induces TLR8 activation and the Th1 type response^34^, and TLR1/2 agonist Pam3Csk4 (Invivogen, 100 nM)^35^, or with pro-inflammatory cytokines (TNF-α-10 ng/ml; IL-6-10 ng/ml; PGE2-1 μg/ml and IL-1β-10 ng/ml), for 48 h. In some experiments, cell-free medium containing Poly (I:C)/LPS was incubated with CNFs (500 μg/ml), or without them for 24 h. The incubation media were then centrifuged at 2000 g for 10 minutes, and the supernatants were collected to treat immature mo-DCs for the following 48 h. All cultures were negative for Mycoplasma, according to the MicoAlert Mycoplasma detection kit (Lonza, Basel, Switzerland). After the cultures, the cells were harvested, washed twice in pure RPMI medium to remove CNFs and stimuli, and then used in the subsequent assays, whereas the mo-DC-culture supernatants were centrifuged at 2000 g for 10 minutes, and then used for the cytokine analysis.

### Flow cytometry

Mo-DCs cultivated with CNFs, or CD4^+^ T cells cultivated with mo-DCs in mixed leukocyte reactions (MLRs), were stained after the culture with the following Abs/reagents: immunoglobulin (Ig)G1a negative control–biotin, IgG1 negative control–PE, IgG1 negative control–FITC, anti-CD86–FITC, anti-HLA-DR-biotin, anti-CD209-biotin, IgG1a negative control-PECy5, anti-CCR7-FITC, anti-ILT3-PE, anti-Foxp3-FITC, anti-CD4-PECy5, anti-TGF-β-biotin, anti-IL-4-PE, anti-CD3-biotin (eBioscience), streptavidin-Alexa 488, anti-CD83-Alexa 488, anti-CD1a-PECy5, IL-17A-PE (Biolegend), streptavidin-PECy5, anti-CD40-FITC, anti-CD25-PeCy5, anti-CD25-PE (BD Pharmigen), anti-IFNγ-FITC, anti-IL-10-FITC (R&D Systems), anti-CD86-PE, CD73-PE (Thermo Scientific), anti-CD4-PE, anti-IL-4-FITC, anti-IL-10-PE (AbD Serotec), anti-CD39-biotin, anti-CD14-FITC (Miltenyi). For surface labeling, the cells were washed once in PBS containing 2% FCS and 0.1% Na-azide and then incubated with primary Abs for 30–60 minutes at 4 °C. Intracellular staining was conducted after the surface staining using the flow cytometry fixation & permeabilisation kit I (R&D). For the intracellular staining of IDO-1, goat anti-human IDO-1 (P20) antibody (Santa Cruz Biotechnology) was used in the fixation & permeabilisation kit I (R&D), as we described previously^20^. Intracellular staining of IFN-γ, IL-4, and IL-17 in CD4^+^T cells, was carried out after the 4 h-activation of T cells with phorbol-12-myristate-13-acetate (20 ng/ml, PMA) and ionomycin (500 ng/ml) in the presence of Monensin (2 μM). Apoptosis was determined after the cultures by staining the cells with PI (10 μg/ml) in a hypotonic citric/Triton-X buffer for 15 minutes, or by Annexin-V-FITC/PI (R&D) labeling, as we described previously^29^. Signal overlap between the FL channels was compensated before each analysis using single labeled cells. Non-specific fluorescence was determined by using the appropriate isotype control antibodies and fluorescence minus one (FMO) controls (Supplementary Fig. 7b).

### Mixed leukocyte reactions

The allostimulatory potential of mo-DCs differentiated with CNFs and control mo-DCs was assessed in MLR with allogeneic CD4^+^ T cells. CD4^+^ T cells (1 × 10^5^/well of 96-well plate) were co-cultivated with different numbers of mo-DCs (1 × 10^4^, 0.5 × 10^4^, 0.25 × 10^4^, 0.125 × 10^4^) for 5 days. The blank controls were mo-DCs and CD4^+^ T cells cultivated separately. Additionally, CD4^+^ T cells were primed with mo-DCs (2 × 10^3^/well of 96-well plate) at a 1/50 mo-DC/CD4^+^ T cell ratio for 3 days, and then Magnetic Activated Cell Sorting (MACS)-purified and expanded with IL-2 (2 ng/mL, R&D) for 2 days. The cells were analyzed for the expression of CD25, FoxP3, CD39, CD73, IL10 and TGF-β, and used in the re-stimulation test or in the suppression assays^20^. The re-stimulation assay included the stimulation of primed CD4^+^T cells with anti-CD3 Ab (5 μg/mL, e-Bioscience) and soluble CD28 Ab (1 μg/mL, e-Bioscience). Proliferation of cells was assessed after 6 days by 3H-thymidine pulsing for the last 18 h (1 μCi/well, Amersham, Books, UK) and the radioactivity was measured by β-scintillation counting (LKB-1219 Rackbeta, Finland). In the suppression assay, MACS purified CD3^+^T cells were labeled with CFSE (2.5 μM, Invitrogen) and were then placed into 96-wells plate (1 × 10^5^ cells/100 μl/well) pre-coated with anti-CD3 Ab (5 μg/mL) and soluble CD28 Ab (1 μg/mL) for 1 h. After that, mo-DC-primed CD4^+^ T cells were added to the co-culture in different numbers (0.5 × 10^5^ − 0.031 × 10^5^ cells/100 μl/well), providing 1:32–1:2 CD4-to-CD3^+^T cell ratios. Controls included cultures of responder CD3^+^T cells, mo-DC-primed CD4^+^T cell and non-stimulated CD3^+^T cells. After 3.5 days, the cells were harvested and analyzed by flow cytometry.

To detect cytokines in the supernatants of primary MLRs and priming assays, parallel mo-DC/CD4+T cell co-cultures were treated with PMA (20 ng/mL) and Ionomicin (500 ng/mL) for the last 4 h, followed by the harvesting of cell-free supernatants. The levels of cytokines produced by mo-DCs, in the primary MLR and the priming assay were measured in cell-free supernatants by sandwich ELISA Kits (R&D).

### Internalization studies

For the light microscopy analyses, the samples were collected from the cultures and the cytospins were prepared and stained with MGG. In some experiments, immature mo-DCs were treated with 7-TOG-MWCNTs (5 μg/ml), which was used as a positive control based on our previous study^15^. Single-cell suspensions were prepared after the culture of mo-DCs with CNFs or 7-TOG-MWCNTs, and the internal complexity of HLA-DR^+^ mo-DCs was analyzed on a flow cytometer by monitoring the side-scatter parameter. The samples for epi-fluorescent microscopy were stained with HLA-DR:biotin/ streptavidin Alexa 488 and Calcofluor. For the confocal microscopy, the samples on cytospins were stained with mouse anti-HLA-DR or anti-CD209, followed by labeling with anti-mouse IgG-Alexa 488. Prior to the analysis with confocal microscope, the samples were stained with Calcofluor. For the TEM analysis, mo-DCs cultivated with CNFs were washed twice in PBS, fixed with 2% glutaraldehyde/PBS, and then post-fixed in 1% OsO4. After dehydration in graded alcohols, thin sections were embedded in Epon 812, sliced on the ultramicrotome, contrasted with lead citrate and uranil acetate, and then analyzed by TME.

### Statistical analysis

Statistical analysis was performed in GraphPad Prism software. Data were not normally distributed according to Shiparo-Wilk test, so Wilcoxon-signed ranked test or Friedman tests with Dunns posttest were performed, depending on whether two or more groups were compared, and values at p < 0.05 or less were considered to be significant statistically.

## Additional Information

**How to cite this article**: Tomić, S. *et al.* Native cellulose nanofibrills induce immune tolerance *in vitro* by acting on dendritic cells. *Sci. Rep.* **6**, 31618; doi: 10.1038/srep31618 (2016).

## Supplementary Material

Supplementary Information

Supplementary Video 1

Supplementary Video 2

Supplementary Video 3
