# Supplementary material for: Native cellulose nanofibrills induce immune tolerance in vitro by acting on dendritic cells
Source: Sci Rep. 2016 Aug 25;6:31618. doi: 10.1038/srep31618 (PMC4997350; doi:10.1038/srep31618)
Supplement: Supplementary Information [file srep31618-s1.pdf]

Native cellulose nanofibrills induce immune tolerance *in vitro* by acting on dendritic cells

Sergej Tomić[1], Vanja Kokol[2], Dušan Mihajlović[1], Aleksandar Mirčić[3], Miodrag Čolić[1,4*]

[1] University of Defense, Medical Faculty of the Military Medical Academy, Belgrade, Serbia

[2] University of Maribor, Faculty of Mechanical Engineering, Institute for Engineering Materials and Design, Maribor, Slovenia

[3] University of Belgrade, Institute of Histology and Embryology, School of Medicine, Belgrade, Serbia

[4] University of Belgrade, Institute for Application of Nuclear Energy, Belgrade, Serbia

*Corresponding author's e-mail: mjcolic@eunet.rs; University of Belgrade, Institute for Application of Nuclear Energy, address: Banatska 31b, 11000 Belgrade, Serbia; Telephone number +381112619252; FAX number +38112618724

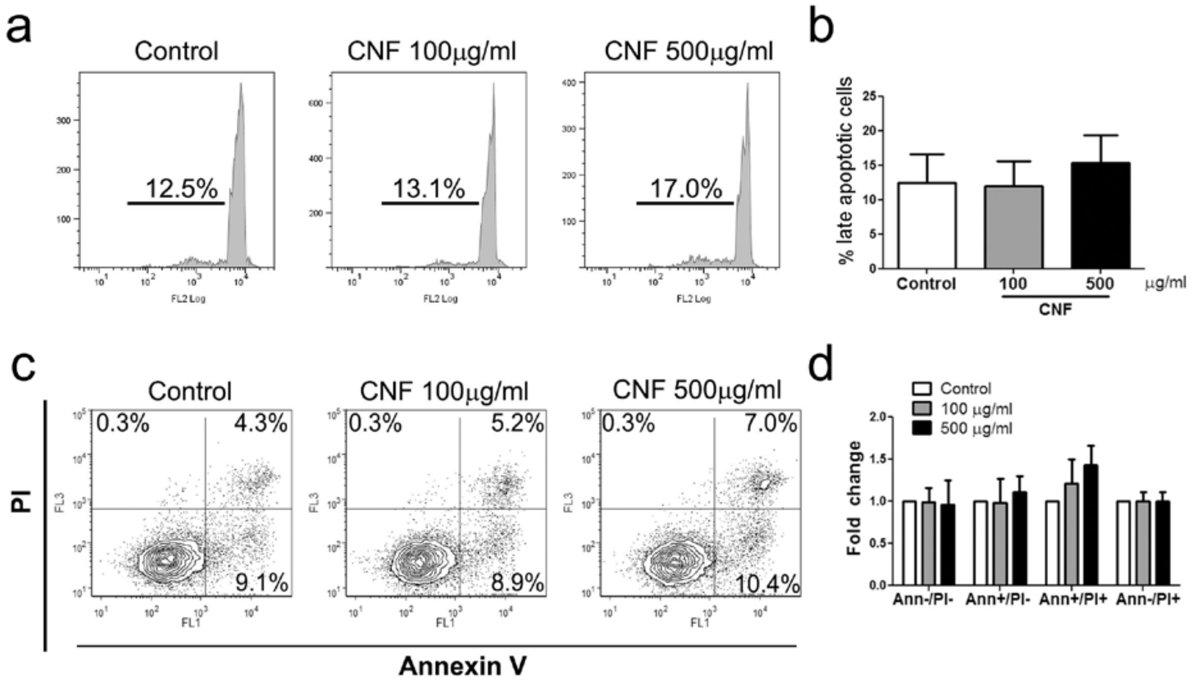

Figure S1. Cytotoxicity of CNFs in culture with DCs. Human monocytes were grown in GM-CSF/IL-4-supplemented medium in the presence of CNFs or their absence (Control) for 5 days, after which the apoptosis was determined by staining the cells with PI in hypotonic solution (a, b) or Annexin-V-FITC/PI staining (c, d). a and b depict representative data obtained by flow cytometry, whereas c and d show summarized results as mean fold change ± SD collected from 4 independent experiments.

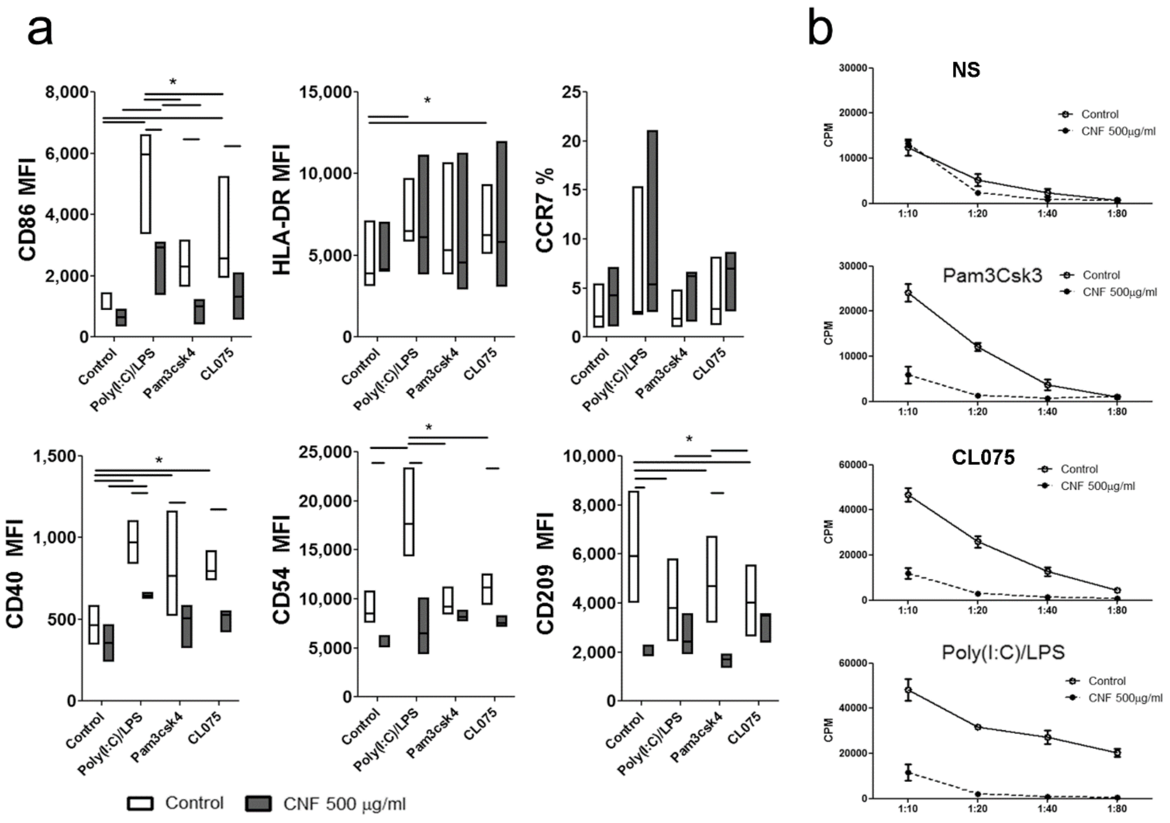

Figure S2. Effects of CNFs on the maturation and allostimulatory capacity of mo-DCs after TLR agonist stimulation. a) The phenotypic analysis of control and CNF-treated mo-DCs was carried out after their stimulation with a combination of Poly (I:C) (25µg/ml) and LPS(100ng/ml), Pam3Csk4 (100nM) or CL075(10µM) or without stimulation (NS), for additional 2 days. The results from 3 experiments with different mo-DCs are shown. *$p<0.05$ as indicated by line (Friedman test with Dunns posttest). b) Representative proliferations detected in co-cultures of mo-DCs and MACS purified $CD4^{+}$T cells ($1x10^{5}$ cells/well), carried out in different mo-DC-to-T cell ratios (1:10-1:80), were measured by 3H-thymidin incorporation assay after 5 days (cpm, counts per minute).

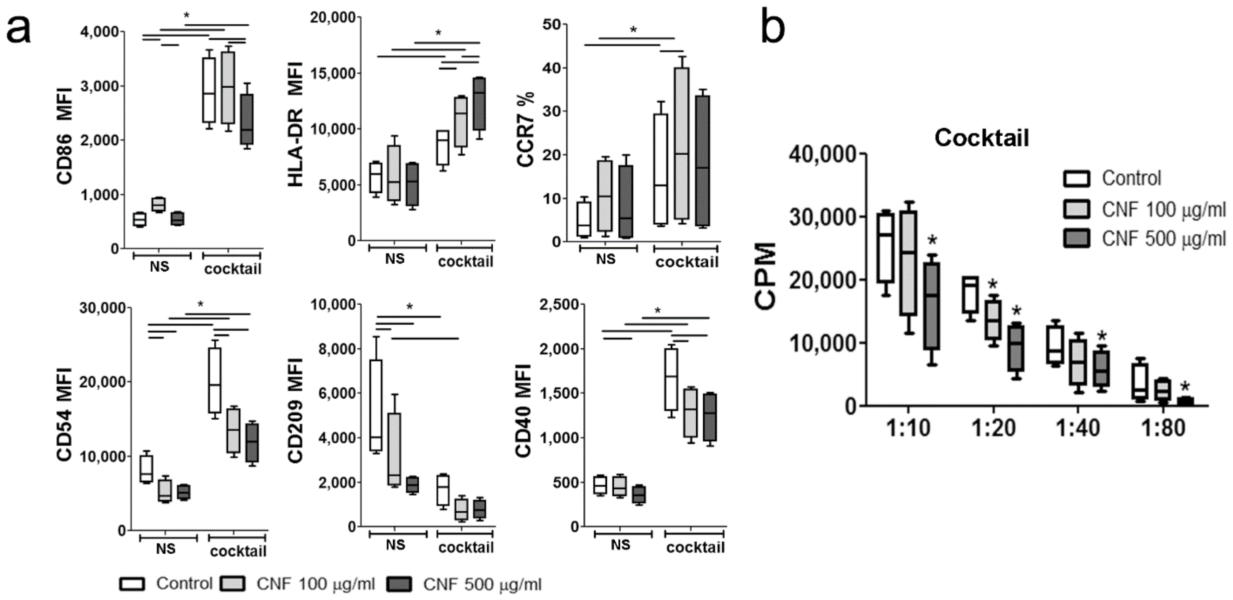

Figure S3. Effects of CNFs on the maturation and allostimulatory capacity of mo-DCs after proinflammatory cytokines stimulation. a) The phenotypic analysis of control and CNF-treated mo-DCs was carried out after their stimulation with a proinflammatory cocktail (TNF-α, IL-6, IL-1β and PGE-2), or without the stimulation (NS), for 2 days. The results from 4 experiments with different mo-DCs are shown. b) The proliferation in co-cultures of proinflammatory cytokines cocktail-treated mo-DCs and MACS purified $CD4^{+}$T cells ($1x10^{5}$ cells/well), carried out in different mo-DC-to-T cell ratios (1:10-1:80), was measured by 3H-thymidin incorporation assay after 5 days (cpm, counts per minute). The results from 4 different mo-DCs/$CD4^{+}$T co-cultures cells are shown. *$p<0.05$ compared to corresponding control, or as indicated by line (Friedman test with Dunns posttest).

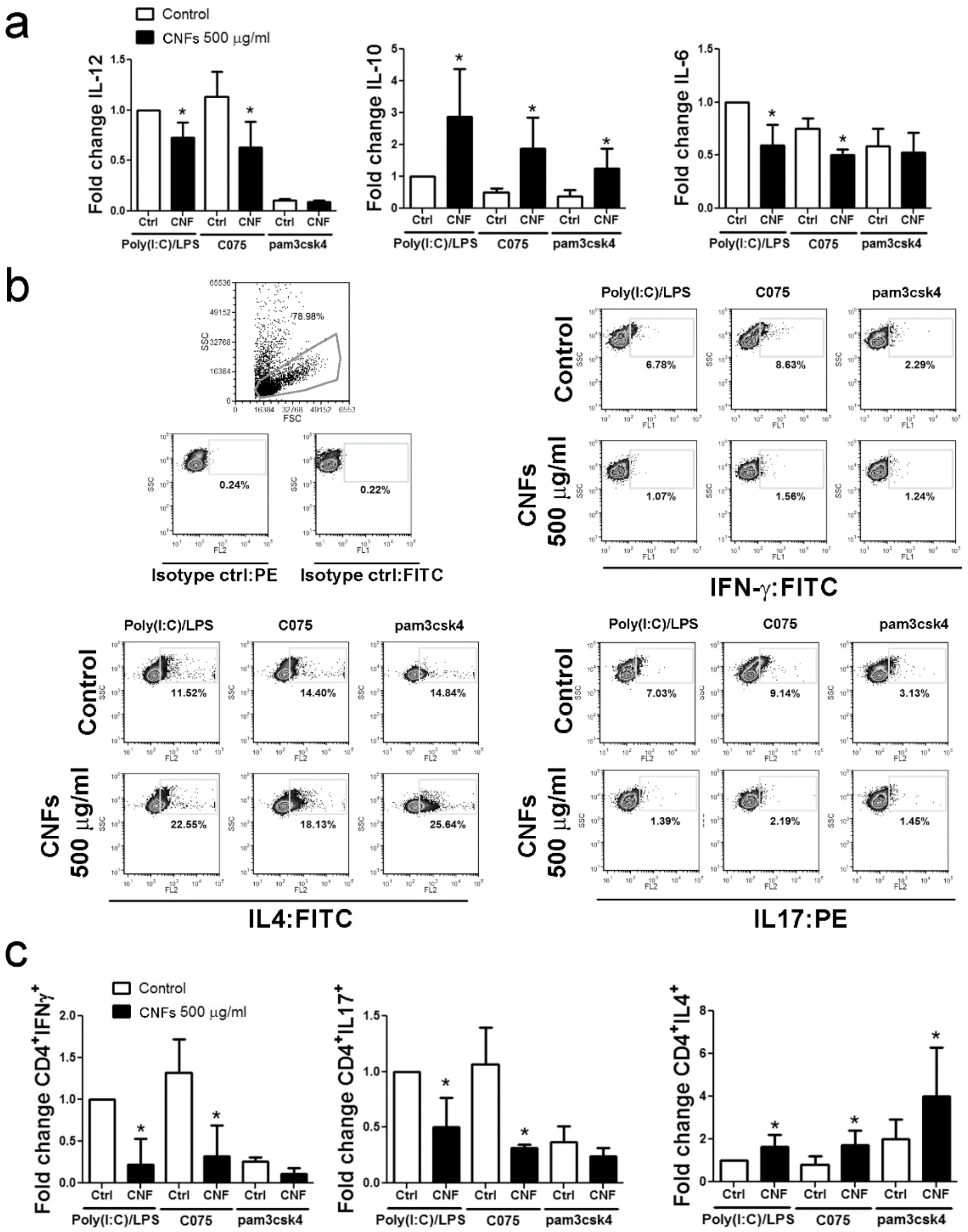

Figure S4. Effects of CNFs on the Th polarization capacity of mo-DCs after their stimulation with TLR agonists. a) The levels of IL-12p70, IL-10 and IL-6 were determined by ELISA in the cultures of CNF (500µg/ml)-treated or control mo-DCs matured with a combination of Poly (I:C) (25µg/ml) and LPS(100ng/ml), Pam3Csk4 (100nM), or CL075(10µM) for 2 days. The results are shown as mean fold change ± SD (n=3 mo-DC donors) of control culture with Poly(I:C)/LPS-treated DCs (index 1) (cytokine: mean ± SD; IL-12: 246.6 ± 126.2 pg/ml, IL-10: 373.3 ± 441.8pg/ml; IL-6: 1395.1 ± 524.1 pg/ml). b) Data from one experiment on intracellular cytokine staining of $CD4^+$T cells co-cultivated with mo-DCs, with low IFN-γ/IL-4 ratio in control cultures, are shown. c) The percentages of IFN-γ^+, IL-4^+ and IL-$17^+$$CD4^+$T cells, from 3 different co-cultures of mo-DCs and $CD4^+$T cell, carried out in 1:20 mo-DC-to-T cell ratio for 5 days, are shown as mean fold change ± SD of control co-culture with Poly(I:C)/LPS-treated DCs

(index 1) (cytokine: mean% ± SD; IFN-γ: 16.6 ± 14.5%; IL-17: 7.8 ± 1.5%; IL-4: 7.5 ± 4.8%) *p<0.05 compared to corresponding control (Friedman test with Dunns posttest).

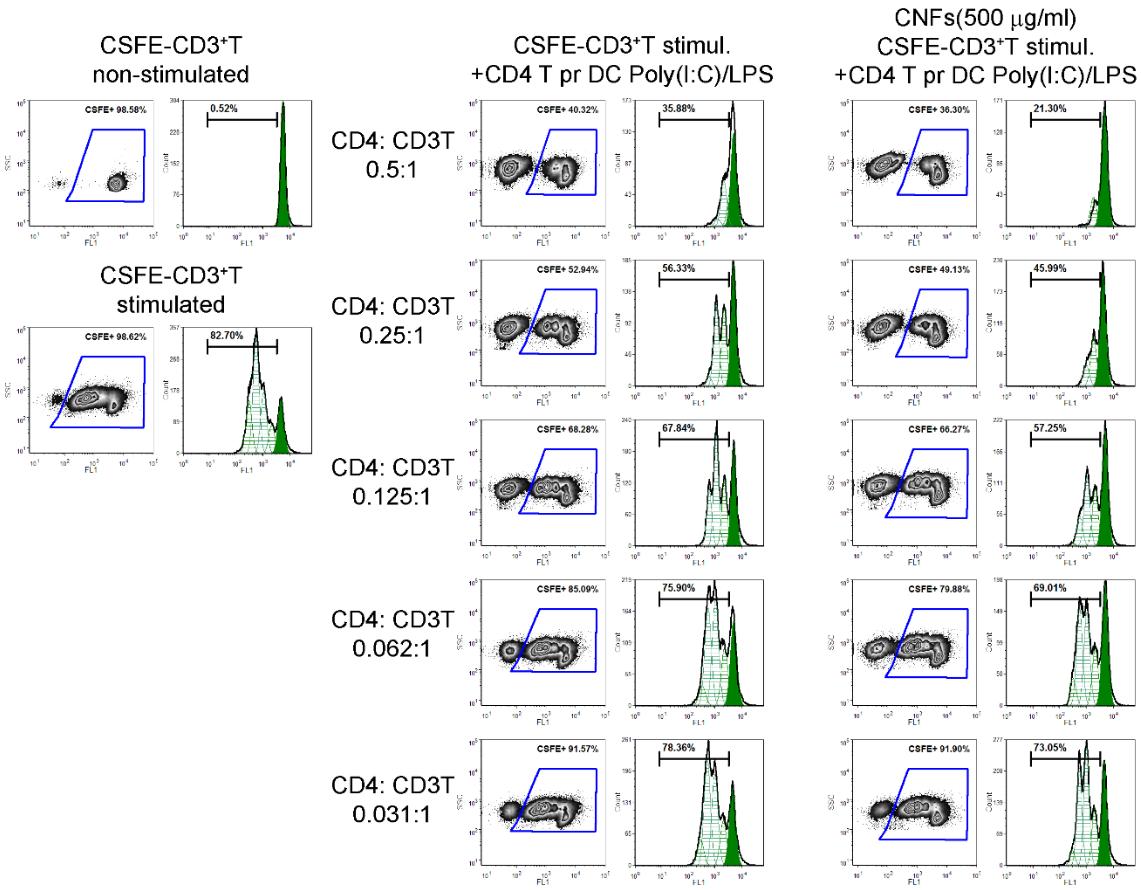

Figure S5. The capacity of mo-DCs-primed CD4$^+$T cells to suppress the proliferation of allogenic CD3$^+$T cells. The responder CFSE-labeled CD3$^+$T cells (1x10^5/well) were added to CD3-coated plates and soluble CD28 for 1h, and after that CD4$^+$T cells that were primed previously with either control or CNF-treated mo-DCs were added in different cell numbers (0.5 - 0.031 x 10^5/well) providing 1:2-1:32 CD4$^+$T:CD3$^+$T cell ratios. The percentage of proliferated responder cells was determined within the CFSE+ gate which separates responder T cells and the non-labeled primed CD4$^+$T cells. A representative experiment is shown out of 3 summarized in Fig. 4.

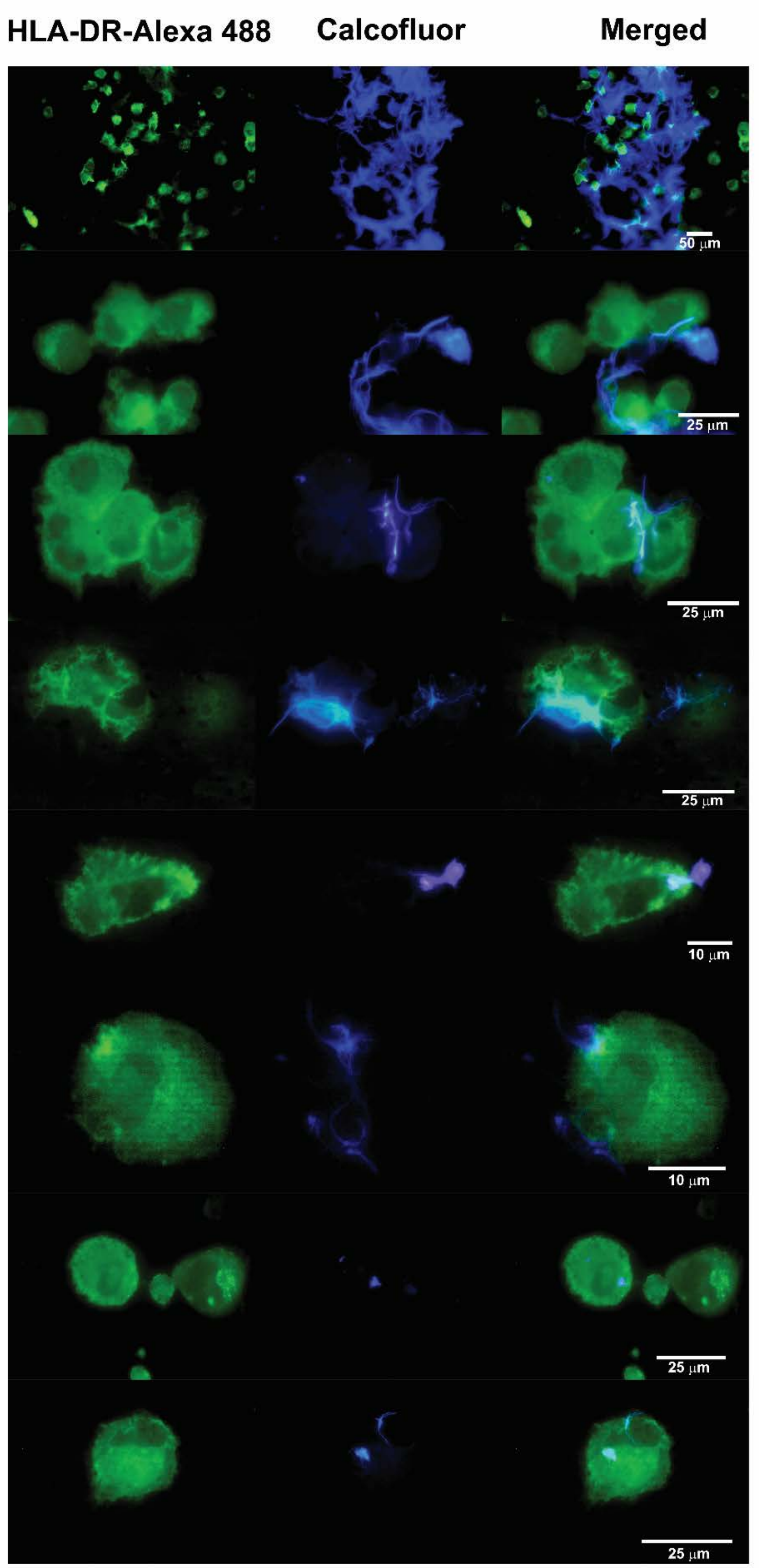

Figure S6. Analysis of DCs and CNFs interactions by epi-fluorescence microscopy. Cytospins of DCs cultivated with CNFs were stained with anti-HLA-DR-Alexa 488 and Calcofluor, and then analyzed by the epi-fluorescent microscopy.

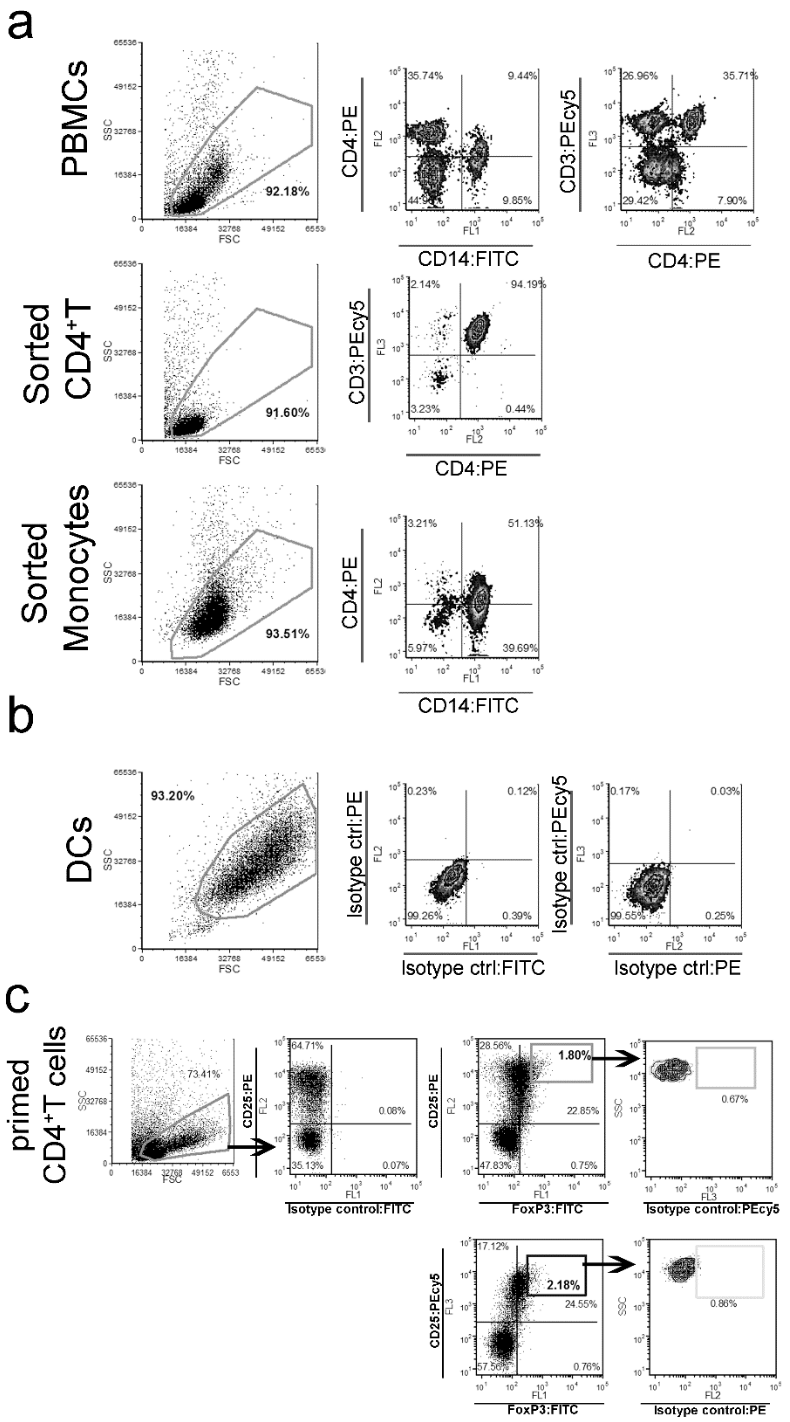

Figure S7. Evaluation of sorted cells and examples of gating strategies. a) CD4^{+}T cells and monocytes were MACS sorted prior to each experiment as shown. b) An example of gating of mo-DCs and corresponding isotype controls are shown. c) Gating and isotype control from Treg analysis (Fig 4e). Single-labeled and FMO controls were used in each experiment to detect a specific fluorescence, as shown in this example.

Supplementary Videos

Video S1. Interaction between large branched CNFs with HLA-DR$^+$ DCs. The samples were stained with anti-HLA-DR Alexa 488 and Calcofluor white, after which Z-stacks were reconstructed in Image J[1] and V3D softwares[2].

Video S2. Interaction between small CNFs with HLA-DR$^+$ DCs. The samples were stained with anti-HLA-DR Alexa 488 and Calcofluor white, after which Z-stacks were reconstructed in Image J[1] and V3D softwares[2].

Video S3. Interaction between large branched CNFs with CD209$^+$ DCs. The samples were stained with anti-HLA-DR Alexa 488 and Calcofluor white, after which Z-stacks were reconstructed in Image J[1] and V3D softwares[2].

Supplemental references

1 Schneider, C. A., Rasband, W. S. & Eliceiri, K. W. NIH Image to ImageJ: 25 years of image analysis. *Nat Methods* **9**, 671-675 (2012).

2 Peng, H., Ruan, Z., Long, F., Simpson, J. H. & Myers, E. W. V3D enables real-time 3D visualization and quantitative analysis of large-scale biological image data sets. *Nat Biotechnol* **28**, 348-353, doi:10.1038/nbt.1612 (2010).
